# The “frosted liver” appearance in hepatic tuberculosis: a rare presentation

**DOI:** 10.1259/bjrcr.20150367

**Published:** 2016-11-02

**Authors:** Neeraj Jain, Harmeet Kaur Rissam, Sunil Kumar Puri, Udit Chauhan

**Affiliations:** Department of Radio diagnosis, Govind Ballabh Pant Institute of Postgraduate Medical Education and Research, New Delhi, India

## Abstract

Tuberculosis is a major re-emerging global health concern. The disease may involve any body system and is a great mimicker of various pathologies owing to its non-specific imaging findings. Herein we report an extremely rare case of atypical tuberculosis of the liver in a young female with complaints of abdominal pain and haematemesis for 10 weeks. Isolated hepatic tuberculosis is a rare entity with < 100 cases reported in the literature. It is therefore important to have a high index of suspicion and be familiar with the atypical imaging findings of abdominal tuberculosis. This discussion highlights the clinical presentation, imaging findings and types of hepatic involvement in tuberculosis.

## Case presentation

A 24-year-old female presented to the gastroenterology department with non-specific abdominal pain for 10 weeks with four episodes of haematemesis during this period. The abdominal pain was mild and non-localized, with no specific aggravating factors. She also reported lack of appetite. She denied any history of fever, cough or weight loss.

Physical examination was unremarkable. No signs of portal hypertension were present. Her haemoglobin, thrombocyte and white blood counts were 7.9 g dl^–1^, 112,000 mm^−3^ and 8010 mm^–3^, respectively, with high lymphocyte count. Erythrocyte sedimentation rate was elevated (112 mm hr^–1^). Human immunodeficiency virus test, serological investigations for hepatitis and cultures of blood and urine were negative. Renal function tests and hepatic transaminase levels were in the normal range. Chest radiograph was normal.

The patient was initially treated symptomatically with antispasmodics and was sent to the radiology department for ultrasonography and abdominal multidetector CT (MDCT) scan to further work-up the aetiology of the abdominal pain and haematemesis.

## Imaging findings

**Figure 1.**
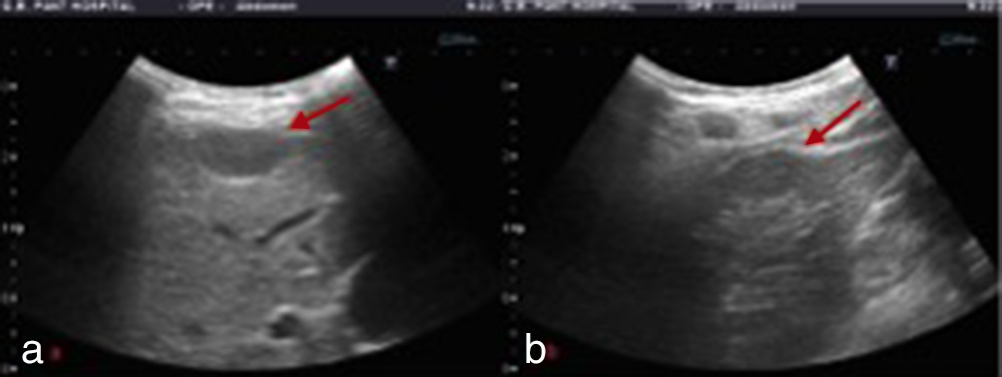
(a, b) Ultrasound images showing hypoechoic, oval-shaped subcapsular hepatic lesions (arrows).

Ultrasonography of the abdomen revealed hepatomegaly with peritoneal thickening in the perihepatic region and multiple, relatively well-defined subcapsular hypoechoic lesions (Figure 1).

**Figure 2.**
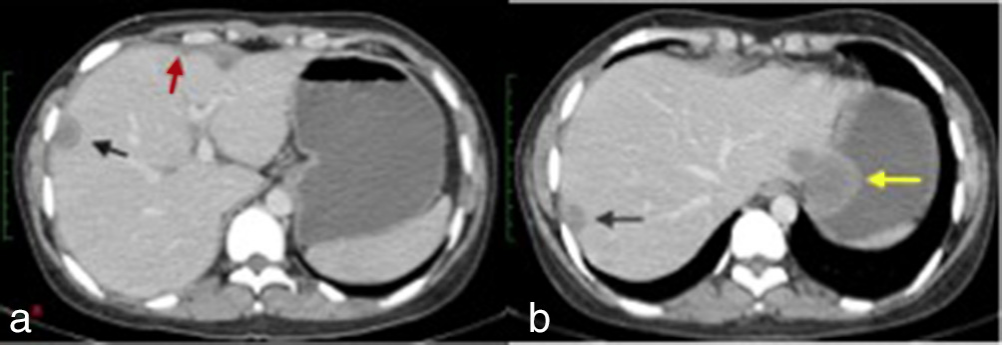
(a, b) Axial contrast-enhanced CT images of the abdomen showing multiple well-circumscribed, hypodense subcapsular lesions (black arrows) in the periphery of the liver with adjacent capsular thickening (red arrow), leading to the “frosted” liver appearance. The largest subcapsular lesion indents are on the gastric fundus (yellow arrow).

A contrast-enhanced CT scan of the abdomen was performed for evaluation of the focal liver lesions. There were multiple well-circumscribed, hypodense subcapsular lesions seen along the periphery of both lobes of the liver. These lesions showed minimal contrast enhancement (Figure 2). The remaining liver parenchyma appeared normal. The largest subcapsular lesion (measuring approximately 39 × 32 mm) in the periphery of the left lobe of the liver (segment II) was exophytic in nature, with indentation on the gastric fundus (Figure 2b). There was no evidence of any internal calcification or fat density in the lesions.

## Histopathological examination and treatment

Ultrasound-guided fine needle aspiration cytology of the subcapsular liver lesion was performed, which revealed a cluster of epithelioid cells with caseous necrosis. A provisional diagnosis of tuberculosis was made and the patient was started on antitubercular therapy. Subsequently, tubercular bacilli were grown on Lowenstein–Jensen medium, which confirmed the diagnosis of tuberculosis. After 6 months of antitubercular treatment, the patient reported symptomatic improvement, and a follow-up ultrasound showed reduction in the size of the lesions.

## Discussion

Hepatic tuberculosis is an uncommon clinical entity and requires a high index of suspicion for diagnosis. Histological or bacteriological findings are required for a definitive diagnosis.^1^ Imaging modalities, particularly MDCT and MRI, are known for their sensitivity in the detection of hepatic nodules and peritoneal involvement. However, owing to the non-specific imaging findings, a multitude of differential diagnosis exist, such as abscesses, metastases and lymphoma.^2^ The Mantoux or tuberculin skin test is of little diagnostic value. The test can be false positive or negative.^3^ Sometimes, the clinical diagnosis of tuberculosis is retrospectively confirmed after recovery post-antitubercular drug therapy.

Some authors have suggested that, in cases lacking aetiological diagnosis of granulomatous hepatitis, patients could undergo an empirical trial with antitubercular drugs, especially in endemic areas of tuberculosis.

Hepatic tuberculosis may occur in primary and secondary tuberculosis, and is particularly common in disseminated tuberculosis. Liver involvement was seen in 80–100% cases of disseminated tuberculosis in an autopsy series,^4^ whereas local hepatic tuberculosis, with minimal or no extra-hepatic manifestations, is much less common.^5^ Kok et al^6^ studied 1678 new tuberculosis cases and reported isolated hepatic tuberculosis in 0.3% of cases. The pathogenesis of these two forms of hepatic tuberculosis is different. Histopathological findings in miliary tuberculosis show that the granulomas are nearly always located inside the lobules, whereas in the local hepatic form, they are mainly found in the portal regions.

The clinical characterization and the nomenclature of isolated hepatic tuberculosis are yet to be clearly defined; this disorder is referred to as atypical tuberculosis of the liver, tuberculous hepatitis, hepatic tuberculosis, hepatobiliary tuberculosis and localized or local hepatic tuberculosis.

The classification of hepatic tuberculosis still remains a subject of debate.^7–9^ Hepatic tuberculosis can be classified according to imaging pattern and pathological findings into parenchymal and serohepatic type, and tuberculous cholangitis. The parenchymal type is the most common among these, and is further divided into three subtypes, that is, miliary, nodular and mixed tuberculosis.

The miliary tuberculosis subtype is the most common form of hepatic tuberculosis. It presents as diffuse, multiple miliary micronodular lesions (2.0 cm in diameter on CT scan), often as a part of tuberculosis in the whole body. Imaging reveals findings of hepatomegaly and micronodular lesions, but it is extremely difficult to find non-calcified lesions < 0.5 cm in diameter with CT scan.

Lesions with diameters > 2 cm characterize nodular tuberculosis. These nodular lesions are easily picked up on imaging; hence, this nodular subtype is the most reported form.^10–12^ Various pathological findings of this subtype are known. In non-caseating tuberculous granulomas or those containing fibrous tissue, a CT scan reveals a hypodense mass with slight peripheral enhancement and is difficult to diagnose correctly. Sometimes there is calcium deposition within the granulomas, giving the appearance of “calcificans punctata”. Marked caseation or liquefaction necrosis leads to the formation of tuberculous abscesses, which appear cystic on MDCT with subtle peripheral enhancement.^10,11^ Coalescing micronodular lesions show the characteristic “cluster” sign.^12^

Mixed tuberculosis, also known as miliary macronodular tuberculosis, has different coexisting pathological stages, including granulomas, liquefaction necrosis, fibrosis or calcification.

Tuberculous cholangitis is rare and is characterized radiologically by irregular and dilated biliary ducts or diffuse calcifications along the course of the bile ducts. It commonly presents as obstructive jaundice and is mainly found in children.

The serohepatic type is the least common type of hepatic tuberculosis; it presents as multiple focal areas of thickened liver subcapsule leading to the “frosted liver” appearance.^7^ The imaging findings of this subtype have been described by Yu et al^13^ in their study of 12 patients with hepatic tuberculosis, wherein they described a patient of serohepatic tuberculosis with multiple hypodense subcapsular liver lesions on contrast-enhanced CT study.

Similar imaging pattern may be seen in some other serious conditions such as lymphoma, metastasis and a variety of infectious/inflammatory and neoplastic conditions; hence, it is important to keep the possibility of tuberculosis in mind when we encounter such cases.^14^

## Conclusions

This case highlights the diagnostic challenge of hepatic tuberculosis. A high index of suspicion is required in order to diagnose serohepatic tuberculosis, as it can present in patients with atypical complaints. Therefore, in patients residing in endemic areas demonstrating capsular liver thickening, hepatic tuberculosis should always be part of the differential diagnosis. This case highlights the importance of the “frosted” liver appearance seen in cases of serohepatic tuberculosis.

## Learning points

1. Hepatic tuberculosis is an uncommon form of gastrointestinal tuberculosis.
2. Hepatic tuberculosis can be classified into parenchymal and serohepatic type, and tuberculous cholangitis. The parenchymal type is the most common among these, and is further divided into three subtypes, that is, miliary, nodular and mixed tuberculosis.
3. The miliary tuberculosis subtype is the most common form of hepatic tuberculosis. It manifests as multiple miliary micronodular lesions (< 2.0 cm in diameter on CT scan), often as a part of tuberculosis in the whole body.
4. The serohepatic type is the least common type of hepatic tuberculosis, seen as multiple focal areas of thickened liver subcapsule leading to the “frosted liver” appearance.

## Consent

Informed consent was obtained from the patient and is held on record.
